# Effectiveness and sustainability of the WHO multimodal hand hygiene improvement strategy in the University Hospital Bouaké, Republic of Côte d'Ivoire in the context of the COVID-19 pandemic

**DOI:** 10.1186/s13756-021-01032-4

**Published:** 2022-02-17

**Authors:** Sophie Alice Müller, Micheline N’Guessan, Rebekah Wood, Lena Landsmann, Carlos Rocha, Bléoué Jean Kouame, Dominique Djadji, Seth Kofi Abrokwa, Tim Eckmanns, Mardjan Arvand, Bamourou Diané, Matthias Borchert

**Affiliations:** 1 Centre for International Health Protection, Robert Koch Institute, Berlin, Germany; 2 University Hospital Bouaké, Bouaké, Ivory Coast; 3 Unit for Evidence-Based Public Health, Robert Koch Institute, Berlin, Germany; 4 Unit for Hospital Hygiene, Infection Prevention and Control, Robert Koch Institute, Berlin, Germany; 5 Unit for Healthcare-Associated Infections, Surveillance of Antibiotic Resistance and Consumption, Robert Koch Institute, Berlin, Germany

**Keywords:** Hand hygiene, WHO multimodal strategy, First WHO Global Patient Safety Challenge, Clean care is safer care, Clean hands, Infection prevention and control, Healthcare-associated infections, Nosocomial infections, Alcohol-based hand rub, AHRB, Local disinfectant production, Bouaké, Côte d’Ivoire, University Hospital

## Abstract

**Introduction:**

The most frequent adverse events in healthcare are healthcare-associated infections, whose burden is highest in resource-limited settings. In addition, low resource settings often lack Hand Hygiene (HH) knowledge and reliable supply to disinfectant, a necessity emphasized by the past West African Ebola Epidemic and the ongoing COVID-19 pandemic. PASQUALE aims to increase patient safety by introducing the WHO multimodal HH strategy in the University Hospital Bouaké, Côte d’Ivoire.

**Methods:**

Assessment of HH knowledge, perception and compliance was performed 12 months before, right after the intervention and at a ten months interval using questionnaires for knowledge and perception and direct observation for compliance. The intervention consisted of a HH training and the introduction of local production of alcohol-based hand-rub. In the absence of a control group, the effectiveness of the intervention was assessed by a before-and-after study.

**Results:**

Baseline knowledge score was 14/25, increased significantly to 17/25 (*p* < 0.001) upon first and decreased to 13/25 in second follow-up. Compliance showed a significant increase from 12.7% to 36.8% (*p* < 0.001) in first and remained at 36.4% in second follow-up. Alcohol-based hand-rub production and consumption almost doubled after first confirmed COVID-19 case in Côte d’Ivoire.

**Conclusion:**

The WHO HH improvement strategy is an effective and pandemic-adaptable method to increase long-term HH compliance. This study emphasizes that the implementation of the strategy to build a robust system is of utmost importance.

**Supplementary Information:**

The online version contains supplementary material available at 10.1186/s13756-021-01032-4.

## Introduction

The most frequent adverse events in healthcare worldwide are healthcare-associated infections (HAIs). Low- and middle-income countries are considered to have two-fold higher HAI rates than high-income countries [1]. As the serious effect of HAIs on patients and healthcare systems is thought to be underestimated in resource limited settings, surveillance and infection-control practices are continuously promoted [2].

The West African Ebola epidemic has led to the reinforcement of infection prevention control (IPC) programs and IPC training protocols [3–5]. These IPC measures emphasize appropriate hand hygiene as the most effective approach to prevent HAI [6]. In 2015, Sierra Leone for example, implemented a National Ebola Recovery Strategy [7] and put in place local production of alcohol-based hand-rub (ABHR) to promote and improve hand hygiene among health care workers [1]. The ongoing COVID-19 pandemic further emphasizes this need for IPC interventions and access to ABHR [8].

However, many healthcare workers (HCWs) in some West African countries including but not limited to Côte d’Ivoire (CI) still lack HH knowledge and reliable access to ABHR [9]. The present study aimed to assess and improve HCW’s HH knowledge and compliance in the University Hospital of Bouaké in order to increase patient safety and evaluate effectiveness and sustainability of the WHO Multimodal Hand Hygiene Improvement Strategy.

## Methods

### Study setting

The University Hospital Bouaké (*Centre Hospitalier Universitaire de Bouaké*, CHUB) is one of five university hospitals in Côte d'Ivoire. The CHUB is a referral hospital and serves a population of more than 500,000 inhabitants. The hospital employs 1087 healthcare workers and has 24 wards. In addition to standard care wards, the CHUB has also some specialized wards such as pediatric surgery or rehabilitation, but no isolation ward yet. The project was implemented hospital wide and supervised by the project team. In terms of ABHR, before the project start mainly soap and water was available for hand washing and some disinfectant gels on ward trolleys. During the COVID-19 pandemic, the CHUB cared for COVID-19 patients as one of the main referral hospitals in Côte d'Ivoire.

The study was conducted as part of the “Partnership to Improve Patient Safety and Quality of Care” project (PASQUALE), responding to the first WHO Global Patient Safety Challenge: “Clean Care is Safer Care” [10]. This partnership was established between the CHUB, the Faranah Regional Hospital, Guinea, and the Robert Koch -Institute, Berlin, Germany.

### Study design

The study was designed as a repeated cross-sectional, uncontrolled before-and-after study consisting of four phases, displayed in the Additional file 1: Fig. S1.

1) baseline assessment, 2) intervention, 3) first follow-up, and 4) second follow-up assessment.

**Phase I: baseline assessment:** In June 2018, a pre-intervention evaluation was conducted including surveys and questionnaires on ward infrastructure, HCW’s perception on HH, and HH knowledge, plus an observation of HH compliance, using tools published by the WHO (Additional files 2–5: questionnaires) [11]. The six major departments (medical, surgery, intensive care, emergencies, obstetrics and pediatrics) were selected and all of the total of 321 HCWs (not including administrative or cleaning staff) were invited to participate. HH compliance was evaluated by direct observation applying the “My 5 Moments for Hand Hygiene” approach of the WHO. This approach defines five indications for a HH action (1) before touching a patient, (2) before clean/aseptic procedures, (3) after body fluid exposure risk, (4) after touching a patient and (5) after touching patient surroundings [12]. The observation of HH practice was carried out by the local hygiene department together with researchers of PASQUALE without prior announcement. All indications and actions were recorded and analyzed following a priority rule which was previously applied in similar settings [13, 14]. This priority rule was applied to ensure that only one indication was associated with each opportunity, specifying a hierarchy for simultaneously occurring indications as follows: before aseptic/clean procedure > after body fluid exposure risk > after touching a patient > before touching a patient > after touching the patient surrounding [15].

**Phase II: intervention**: Prior to the HH training, there was the need to set up a reliable and regular supply of ABHR by establishing a local production unit. In June 2019, a team of six including experts from the laboratory, hygiene department and pharmacy was formed to produce ABHR in CHUB, after attending a four-day workshop for local production. The workshop also included onsite training and was conducted by an international expert on local production according to the WHO guidelines for “formulation 1” ABHR, consisting of ethanol 80% (v/v), glycerol 1.45% (v/v), and hydrogen peroxide 0.125% (v/v) [14, 16]. To enhance south-south exchange within the partnership, the local production team from the Regional Hospital of Faranah, Guinea assisted in this training, leading to a stronger network for local ABHR production advice and support. For example, partners contacted each other in case of supply shortage of raw materials or questions regarding stocking procedures. To facilitate local production for the entire CHUB a manufacturing room was constructed. The production schedule was based on a needs assessment conducted by the local hygiene team. Upon request of the production team a partial efficacy testing was carried out in the laboratories of the unit for Hospital Hygiene, Infection Prevention and Control at the Robert Koch Institute, using a suspension test according to the European Norm DIN EN 13,727 with *Enterococcus hirae* as test organism. No intolerance of ABHR was reported during the project duration.

After ABHR had been distributed, a HH training for HCWs was conducted in August 2019. This training was adapted to the needs identified in the baseline assessment. Preliminary results from the baseline assessment were presented to the HCW. The face to face training included didactic and practical sessions. The training was held as a one-day workshop and took place on four occasions for a minimum of four participants of every department.

The local coordination team gave equal access to the HH training to all CHUB departments regardless of their participation in the baseline assessment. Approximately four members of each department, regardless whether the department is part of the selected six main wards, were invited. This was meant to provide exposure to new knowledge to representatives of all departments, so that this information could be further spread within their respective department. In terms of research, this led to unequal distribution of professional groups between different assessment phases, as medical/nursing students participated in the baseline assessment only, while administrative and cleaning staff-members participated in the first follow-up but not in the baseline. All groups are shown in the description of the study population (Table 1), but unequally represented groups are excluded from further data analysis in order to compare like with like. In conjunction with the training, WHO posters and flyers on HH were distributed throughout the wards and were available throughout the remainder of the study period.

**Table 1:** Study population participating in questionnaires

	Baseline N (%)	1st Follow-up N (%)	2nd Follow-up N (%)
Number of Respondents	218	149	162
Sex (female)	110 (50.5)	65 (43.6)	82 (50.6)
Age group (years)			
20–39	159 (72.9)	82 (55.0)	99 (61.1)
40 +	57 (26.1)	66 (44.2)	62 (38.3)
Missing	2 (0.9)	1 (0.7)	1 (0.6)
Respondents by profession			
Medical doctor	56 (25.7)	22 (14.8)	48 (29.6)
Nurse	31 (14.2)	33 (22.2)	35 (21.6)
Midwife	26 (11.9)	4 (2.7)	30 (18.5)
Auxiliary Nurse	45 (20.6)	33 (22.2)	26 (16.1)
Nursing Student*	21 (9.6)	0	0
Medical Student*	35 (16.1)	0	8 (4.9)
Technician*	0	15 (10.1)	0
Pharmacy*	0	3 (2.0)	0
Physiotherapist*	0	1 (0.7)	0
Hygienist*	0	8 (5.4)	0
Cleaner*	0	6 (4.0)	0
Administrative*	0	9 (6.0)	0
Others*^#^	4 (1.8)	15 (10.1)	15 (9.3)

^*^Excluded from further analysis because of grossly uneven representation in the assessments;

^#^according to the WHO description: dietician, dentist, social worker, etc.

**Phase III: first follow-up assessment:** From August 2019 to November 2019, a post-intervention evaluation was undertaken. This evaluation consisted of a follow-up assessment of HH knowledge and perception questionnaires directly after the training, including the staff that has participated in the training. HH Compliance was observed in the following month until November 2019 in the six main departments identified in phase I only. Furthermore, monthly production of ABHR and consumption was tracked from July 2019 until the end of 2020. The local production team was encouraged to informally explore HCW attitudes towards the ABHR.

**Phase IV: second follow-up assessment:** To assess long-term effects, a second post-intervention evaluation on HH knowledge and compliance was conducted ten to twelve months after the intervention from June to August 2020 in the six main wards. The study team refrained from re-assessing perception for logistical reasons linked to the COVID-19 pandemic.

### Statistical analysis

All data was entered in WHO Epi Info data templates and analyzed using Stata 15.2 (StataCorp LLC, College Station, Texas, USA). As part of data entry quality assurance, all data was doubled entered by two research assistants and plausibility checks applied.

A knowledge score was calculated based on the HH knowledge questionnaire responses, equaling the number of correct answers (maximum score 25 points). Wilcoxon rank-sum test was performed to assess differences between median scores at different project phases. To assess the association between the intervention and knowledge score, a multiple linear regression was performed controlling any confounding effect of gender, age group, profession and ward. HCWs perception on the five components of the WHO multimodal HH Strategy was assessed in baseline and follow-up questionnaires.

Post-intervention perception was reported as the percentage of follow-up respondents answering “seven” on a seven-point Likert scale, where 1 equaled “not effective” and 7 “very effective”.

HH compliance was calculated as the number of performed HH actions divided by all opportunities requiring HH actions according to the WHO 5 Moments of HH. Baseline and follow-up compliance were compared using the χ^2^ test. A multivariable logistic regression was performed with compliance as the outcome, period (pre-/post-intervention) as the main independent variable and confounders proposed in the literature, such as profession, department and indication group [17]. All confounders were included in the initial model and maintained if the crude OR differed substantially from the adjusted one.

*p*-values less than 0.05 were considered as statistically significant; when applicable the two-tailed variety of the test was used. The compliance observations were not independent as more than one HH opportunity per HCW was usually observed without individual identification. To account for the lack of independence, we followed the approach of a previous study [18], applying a design effect of two and consequently doubling the standard error [14].

## Results

A total of 218, 149 and 162 HCW participated in the assessment of HCW’s knowledge and perception in baseline, first and second follow-up respectively. Professions were divided into thirteen groups, twelve being specific professional categories and one category “others”.

Since all staff were given equal chance to participate in the 1st follow-up, the professional composition of the study population differed between phases. The categories “nursing student, medical student, pharmacy, physiotherapist, hygienist, cleaner, administrative and others” were grossly unevenly represented and therefore excluded from further analysis.

### Hand hygiene knowledge

30.7% of HCWs reported to have had a HH training within the last three years. The median knowledge score was 14.0 (IQR 13.0–16.0) out of a maximum of 25 in baseline, which significantly increased to 17.0 (IQR 15.0–19.0) in first follow-up (*p* < 0.001) and significantly decreased to 13.0 (11.0–15.0) in second follow-up (*p* < 0.001) (Table 2). The knowledge increase upon first follow-up was considerable and significant in all professional groups except in “Auxiliary nurses” (“Medical Doctors” + 4, *p* < 0.001; “Nurse” + 4, *p* < 0.001; “Midwives” + 5, *p* = 0.004; “Auxiliary Nurse” + 1, *p* = 0.432) This knowledge increase was not maintained in 2nd follow-up compared to 1st follow-up (“Medical Doctors” − 4, *p* < 0.001; “Nurse” − 4, *p* < 0.001; “Midwives” − 7, *p* = 0.002; “Auxiliary Nurse” − 2, *p* < 0.001). In 2nd follow-up all professional groups fell back to baseline knowledge level or below (Table 2). Multiple linear regression found no major confounding by gender, age group, profession or ward, and showed a significant association between the intervention and HH knowledge in first follow-up (regression coefficient of 2.6; 95% CI 2.0–3.2; *p* < 0.001).

**Table 2:** Median hand hygiene knowledge score (IQR), maximum score: 25

	Baseline	1st Follow-up	p**	2nd Follow-up	p*	p**
Overall Knowledge Score	14.0 (13.0–16.0)	17.0 (15.0–19.0)	< 0.001	13.0 (11.0–15.0)	< 0.001	< 0.001
By professional categories						
Medical doctor	15.0 (13.5–16.5)	19.0 (17.0–22.0)	< 0.001	15.0 (13.0–17.0)	< 0.001	0.828
Nurse	14.0 (14.0–16.0)	18.0 (17.0–20.0)	< 0.001	14.0 (11.0–15.0)	< 0.001	0.057
Midwife	14.0 (13.0–16.0)	19.0 (18.0–19.5)	0.004	12.0 (10.0–14.0)	0.002	0.004
Auxiliary nurses	14.0 (13.0–15.0)	15.0 (14.0–17.0)	0.432	13.0 (10.0–14.0)	< 0.001	0.015

^*^p-value calculated with Wilcoxon rank-sum test compared to 1st follow-up

^**^p-value calculated with Wilcoxon rank-sum test compared to baseline

### HCWs’ perception of the strategy components

The WHO multimodal HH strategy and its impact were positively perceived in baseline and follow-up. Over 87.4% of respondents considered HH to be “highly” or “very highly” effective to prevent HAI in baseline and follow-up. However, only 20.8% rated the hypothetical invitation of patients to remind HCW to perform HH to be effective in baseline and 34.2% in first follow-up.

HCWs reported themselves to be compliant with HH according to the 5-moments in at least 64.0% of overall indications at baseline and in 59.0% in follow-up. The questionnaire on the perceived impact of the project intervention showed that training and the availability of ABHR were rated to have the highest impact on HH improvement, while the feedback of the results from the compliance observation was felt to have the lowest impact (Table 3).

**Table 3:** HCWs’ perception about impact of intervention

	1st Follow-up N (%)*
Has the use of ABHR made hand hygiene easier to practice in your daily work?	118 (79.2)
Is the use of ABHR well tolerated by your hands?	108 (73.0)
Did knowing the results of hand hygiene observation in your ward help you to improve your hand hygiene practices?	88 (61.1)
Has the fact of being observed made you paying more attention to your hand hygiene practices?	88 (69.9)
Were the educational activities that you participated in important to improve your hand hygiene practices?	131 (87.9)
Has the improvement of the safety climate (…) helped you personally to improve your hand hygiene practices?	100 (67.1)
Has your awareness of your role in preventing HAIs by improving your hand hygiene practices increased during the current hand hygiene promotional campaign?	107 (71.8)

^*^results are shown as number of respondents out of total selecting seven on a seven-point Likert scale, (1 = “not effective”; 7 = “very effective”)

### Compliance with hand hygiene

In total 2475 HH opportunities were observed (baseline 719, 1st follow-up 921, 2nd follow-up 835). All reported *p*-values refer to the comparison with baseline, as differences between 1st and 2nd follow-up were all small and not significant.

**Fig. 1:**
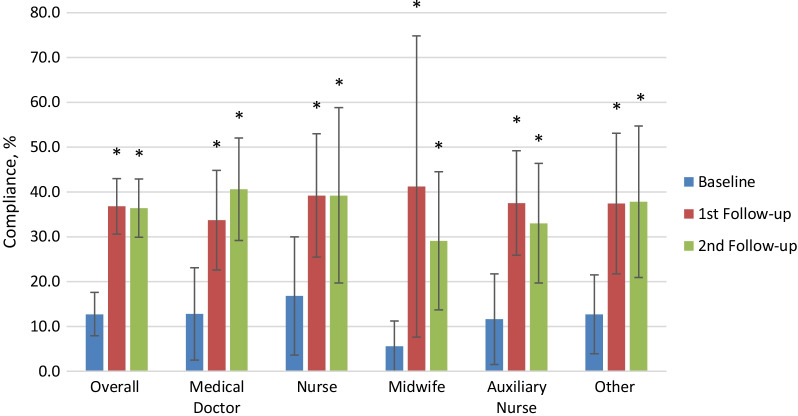
Hand Hygiene Compliance at Baseline, 1st and 2nd Follow-up, overall and by profession. Overall compliance: baseline compared to 1st follow-up *p* < 0.001, baseline compared to 2nd follow-up *p* < 0.001, 1st follow-up compared to 2nd follow-up *p* = 0.001. * if *p* < 0.05 compared to baseline. Error bar presents 95% confidence interval (CI). *p*-values and CI were adjusted for lack of independence by inflating the standard error by a factor of 2. CI are restricted to positive numbers and values up to 100%

**Fig. 2:**
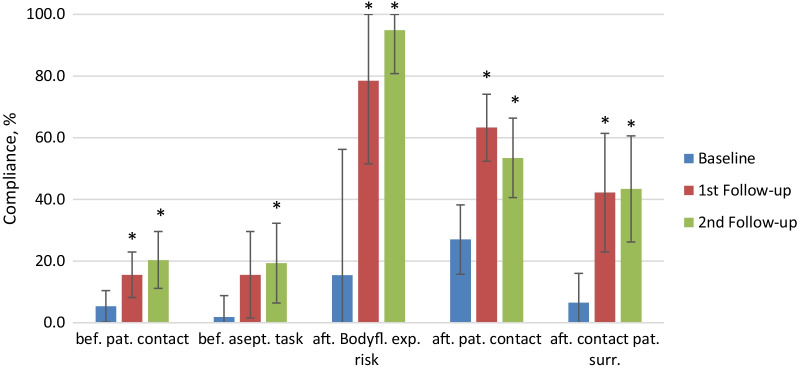
Hand Hygiene Compliance at Baseline, 1st and 2nd Follow-up, by indication. *If *p* < 0.05 compared to baseline. Error bar presents 95% confidence interval (CI). *p*-values and CI were adjusted for lack of independence by inflating the standard error by a factor of 2. CI are restricted to positive numbers and values up to 100%

The overall compliance at baseline was 12.7% and increased significantly (*p* < 0.001) to 36.8% at first follow-up and leveled at 36.4% at second follow-up (*p* < 0.001, Fig. 1). All professional groups showed a significant increase in compliance in the first follow-up (“Medical Doctors” + 20.9, *p* = 0.001; “Nurse” + 22.4, *p* = 0.003; “Midwives” + 35.6, *p* = 0.004; “Auxiliary Nurse” + 25.9, *p* < 0.001; “Others” + 24.7, *p* < 0.001). This significant increase compared to baseline was maintained at second follow-up (“Medical Doctors” + 27.8, *p* < 0.001; “Nurse” + 22.4, *p* = 0.008; “Midwives” + 23.5, *p* = 0.013; “Auxiliary Nurse” + 21.4, *p* = 0.001; “Others” + 25.1, *p* < 0.001). Compliance rose considerably in the first follow-up across all indications (Fig. 2); this improvement was significant except for “before aseptic task” showing a borderline result only (*p* = 0.058). Compliance across all indications continued to rise in the second follow-up with the exception of “after patient contact”, which dropped by 10 percentage points. The indication “after body fluid exposure risk” had the largest overall increase, reaching the best compliance among all indications upon second follow-up (95%). “Before aseptic task” had the lowest baseline compliance rate but steadily increased significantly (*p* = 0.025) to 19.3% upon second follow-up. By the 2nd follow-up, the compliance levels observed for “before patient contact” indication were considerably and significantly lower than for “after patient contact” indication (20.3% vs. 53.5%, *p* < 0.001).

Between first and second follow-up no significant differences were observed. For more detailed analysis please see Additional file 6: Table S1.

Multivariable analysis showed that the increase in compliance in the first follow-up was associated with the intervention (crude OR of 4.0; 95% CI 2.4–6.7; *p* < 0.001). This association remained significant and became stronger after adjusting for the confounding factor of indication (adjusted OR of 5.2; 95% CI 2.9–9.1; *p* < 0.001).

### Production and consumption of ABHR

Local production of ABHR in the hospital pharmacy was launched in July 2019 with regularly scheduled production sessions. In average, 74.4L per month were produced from July 2019 until March 2020, when the first COVID-19 infection was confirmed in Ivory Coast [19], and 138L per month from April 2020 onwards. In three independent experiments, efficacy testing of the locally produced ABHR revealed ≥ 5 log10 reduction of *E. hirae*, fulfilling the requirements of the European Norm DIN EN 13727 for this test organism for successful disinfection. However, the local ABHR production team noticed hesitation in using the locally produced ABHR prior to the pandemic. Reasons given by the HCW to the local production team through informal discussions were that HCW reported lack of confidence in the quality of the local product in comparison to the commercial product imported from France; desire for a gel-based product; requests for perfume additives, and finally wish for improvement of the bottle label design. Despite these potential shortcomings, the monthly consumption of ABHR increased from 76L prior to the pandemic to 125L after March 2020.

## Discussion

HH compliance increased considerably and significantly across all professional categories and was maintained ten to twelve months after the intervention. A large and significant increase was also shown in knowledge in first follow-up. However, this was not long lasting. The second follow-up found a large decrease compared to first follow-up and a small but significant decrease when compared to baseline. The latter could be partly explained by the fact that the study populations were not the same at baseline and follow-up, and that the transfer of knowledge from HCW to HCW had not taken place to the extent as expected. The low knowledge could also be a result of the change in circumstances, which could have rendered the assessment of knowledge more difficult. However, this low knowledge was not expected given the worldwide promotion of HH in the context of the COVID-19 pandemic. The COVID-19 pandemic has challenged healthcare systems and might have led to lack of time for investing in assessments. This rationale is supported by numerous uncompleted questionnaires and by a known high stress level of healthcare workers during the current pandemic [20–22].

The compliance in first follow-up (36.8%) was threefold the baseline compliance and was higher than compliance at follow-up in the comparable settings of a referral hospital in Mali (21.8%) and a university hospital in Ethiopia (11.7%) [14, 23]. This improvement matched the pattern at the PASQUALE partner hospital in Guinea, which also had a threefold increase of compliance in first follow-up [24]. In the second follow-up, the increased compliance level was maintained except for one indication. So far, the development of compliance has triphasic learning curve which literature describes as a rapid initial learning phase (right after the intervention), accompanied by a decline in the improvement, and followed by a recovery to a steady state of improvement [25]. This triphasic curve has been observed in other studies including the PASQUALE partner hospital in Guinea (manuscript under review). This maintained increase could partially be attributed to the ongoing COVID-19 pandemic, as healthcare workers are known to be concerned about the spread of infection [26], which may have motivated them to maintain their compliance at a relatively high level. In addition, we would recommend steps to further increase compliance such as regular refresher trainings, fostering of HH culture through HH championship and facilitation of patient involvement. Patient involvement, however, can be challenging in hospital settings where paternalistic HCW patient dynamics may dominate, and at CHUB only a minority of HCWs rated the inclusion of patients to be an effective HH measure. The indication of “before aseptic procedure” had the lowest baseline compliance of 1.8%, being ten times lower than in nurses of an Ethiopian University Hospital [27], and much lower than the Guinean partner hospital baseline of 11.4% [24]. This compliance increased to only 15.5% in first follow-up and remained lowest in second follow-up. This level is alarmingly low, considering that this indication is particularly important for preventing HAIs, especially catheter-related bloodstream infections (CRBSI) and that it may have the greatest impact on patient safety [28]. The second lowest compliance was observed in the indications “before patient contact”, being much lower compared to “after patient contact”. This result is in line with most of HH compliance studies and could indicate that HCWs focussed on protecting themselves rather than patients [29]. A remarkable compliance increase from 15% via 78% to 95% in the indication “after body fluid exposure risk” can again be explained by the rationale that self-protection was particularly important to HCWs, and that this attitude was intensified by the COVID-19 pandemic in the second follow-up, since COVID-19 can be transmitted through respiratory droplets and possibly also other body fluids [30]. Future trainings should help raise awareness among HCWs of the discrepancy between perceived and implemented HH actions, as self-assessment showed two times higher rates of perceived compared to observed application. In addition, there is an urgent need to raise awareness among healthcare workers about their own protection and, at the same time, about their duty to prevent infections such as CRBSI and ensure patient safety. Local production is considered to be a cost-effective measure to improve HH, as it had already been demonstrated by modelling [31] and a quasi-experimental study [32]. Local production is also flexible to respond to increasing needs. The local production team was able to increase the production more than 100% during the ongoing COVID-19 pandemic. The worldwide stockouts and export disruptions of disinfectants further emphasized the advantages of local ABHR production, making supplies rather independent from national and global shortages. These advantages had been anticipated by the local partners when they constructed a dedicated manufacturing room for local ABHR production and storage even prior to the pandemic.

Before the COVID-19 pandemic had reached Bouaké, the local production team reported HCWs’ hesitation of using the locally produced ABHR due to lack of confidence in the product and desire for additional features. The pandemic alleviated this concern, as ABHR consumption almost doubled after the first confirmed COVID-19 case in the CHUB in Côte d’Ivoire. Nevertheless, we plan to explore this hesitation in a qualitative study to develop strategies to enhance the product’s attractiveness together with the local HCWs.

One limitation of the study was that the influence of the current pandemic could not be measured or compared to previous studies in this setting. At the same time, we could collect insights on how the pandemic may have impacted on HH behaviour. Another limitation of the present study is the lack of relating HAI rates to the HH intervention. Assessing HAI rates by clinical departments in collaboration with the CHUB laboratory could be a valuable possibility for assessing the impact of HH improvement, as well as for information sharing and interdisciplinary collaboration. Furthermore, giving equal access to the training for all hospital wards affected the comparability of the study populations at the different project phases, which was considered by excluding the group “other” in the analysis. We cannot exclude the possibility of selection bias and hence the over-estimation of knowledge as HCWs with lower motivation and HH knowledge could be less likely to participate, but over 30% and 50% of HCWs of the six main wards participate in baseline and in the 2nd respectively. In terms of HH compliance, giving access to all HCWs appears to have been successful considering its sustained improvement.

## Conclusion

The WHO HH improvement strategy is an effective and pandemic-adaptable method to sustainably increase HH compliance in resource limited settings. This study emphasized that the implementation of a HH strategy prior to an epidemic is of utmost importance to improve the independence and responsiveness to the epidemic.

## Supplementary Information

**Additional file 1**. Timetable.

**Additional file 2**. WHO Observation Form.

**Additional file 3**. WHO Ward Infrastructure Survey.

**Additional file 4** WHO Hand Hygiene Knowledge Questionnaire for Health-Care Workers.

**Additional file 5**. WHO Perception Survey for Health-Care Workers.

**Additional file 6**. Hand Hygiene Compliance at Baseline and Follow-up.

## Data Availability

The datasets generated and/or analysed during the current study are not publicly available due ethical and data protection reasons, but are available from the corresponding author on reasonable request. The data contain potentially identifying information: our data have been collected from a small group of participants, and even data that are not directly identifying in combination become identifying (e.g. sex, profession, ward).

## Abbreviations

ABHR: Alcohol-based hand-rub
CHUB: Centre Hospitalier Universitaire de Bouaké
HCW: Healthcare worker
HH: Hand hygiene
HAI: Healthcare-associated infections
IPC: Infection prevention and control
IQR: Interquartile range
OR: Odds ratio
PASQUALE: Partnership to Improve Patient Safety and Quality of Care
WHO: World Health Organization
