# Supplementary material for: Effectiveness and sustainability of the WHO multimodal hand hygiene improvement strategy in the University Hospital Bouaké, Republic of Côte d'Ivoire in the context of the COVID-19 pandemic
Source: Antimicrob Resist Infect Control. 2022 Feb 17;11:36. doi: 10.1186/s13756-021-01032-4 (PMC8851710; doi:10.1186/s13756-021-01032-4)
Supplement: Supplementary file 1 — Additional file 1. Timetable. [file 13756_2021_1032_MOESM1_ESM.pptx]

## Slide 1
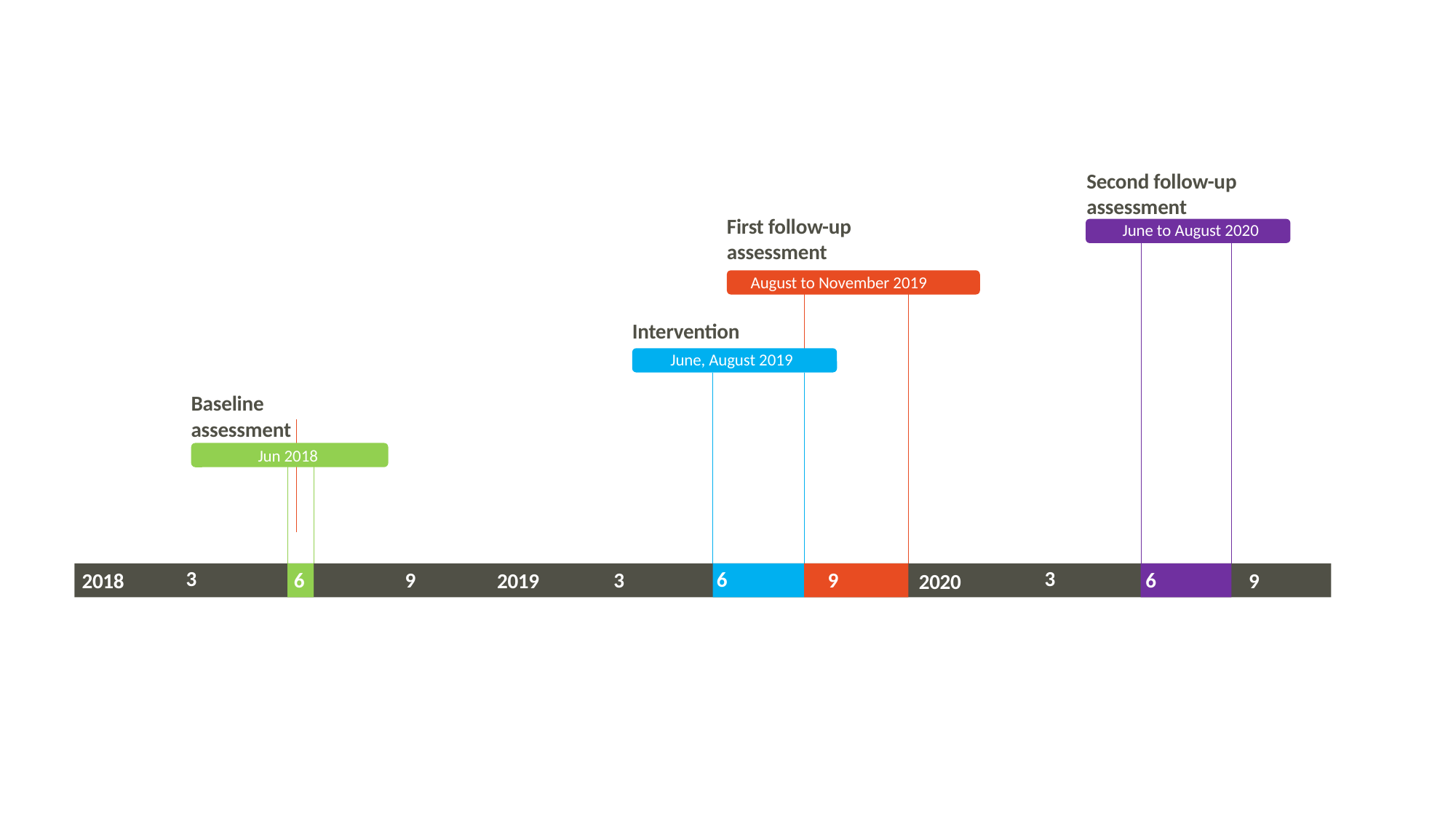

Second follow-up assessment
First follow-up assessment
June to August 2020
August to November 2019
Intervention
June, August 2019
Baseline assessment
Jun 2018
3
3
6
6
6
3
9
9
9
2019
2018
2020
Today
Mar 31
Jun 30
Sep 30
2020
2022
