# Supplementary material for: Effectiveness and sustainability of the WHO multimodal hand hygiene improvement strategy in the University Hospital Bouaké, Republic of Côte d'Ivoire in the context of the COVID-19 pandemic
Source: Antimicrob Resist Infect Control. 2022 Feb 17;11:36. doi: 10.1186/s13756-021-01032-4 (PMC8851710; doi:10.1186/s13756-021-01032-4)
Supplement: Supplementary file 2 — Additional file 2. WHO Observation Form. [file 13756_2021_1032_MOESM2_ESM.doc]

Observation Form

| **Facility:** |  | **Period Number*****:** |  | **Session Number*****:** |  | |
| --- | --- | --- | --- | --- | --- | --- |
|  |  |  |  |  |  |  |
| **Service:** |  | **Date:  (mm/yy)** | / | **Observer:**  **(initials)** |  | |
|  |  |  |  |  |  |  |
|  |  |  |  |  |  |  |
| **Department:** |  | **Session duration: (mm)** |  | **City******:** |  | |
|  |  |  |  |  |  | |
| **Country******:** |  |  |  |  |  | |

| **Prof.cat** | |  | | **Prof.cat** | |  | | **Prof.cat** | |  | | **Prof.cat** | |  | |
| --- | --- | --- | --- | --- | --- | --- | --- | --- | --- | --- | --- | --- | --- | --- | --- |
| **Code** | |  | | **Code** | |  | | **Code** | |  | | **Code** | |  | |
| **N°** | |  | | **N°** | |  | | **N°** | |  | | **N°** | |  | |
| **Opp.** | **Indication** | | **HH Action** | **Opp.** | **Indication** | | **HH Action** | **Opp.** | **Indication** | | **HH Action** | **Opp.** | **Indication** | | **HH Action** |
| **1** | bef-pat.  bef-asept.  aft-b.f.  aft-pat.  aft.p.surr. | | HR  HW  🌕 missed  🌕 gloves | **1** | bef-pat.  bef-asept.  aft-b.f.  aft-pat.  aft.p.surr. | | HR  HW  🌕 missed  🌕 gloves | **1** | bef-pat.  bef-asept.  aft-b.f.  aft-pat.  aft.p.surr. | | HR  HW  🌕 missed  🌕 gloves | **1** | bef-pat.  bef-asept.  aft-b.f.  aft-pat.  aft.p.surr. | | HR  HW  🌕 missed  🌕 gloves |
|  |  | |  |  |  | |  |  |  | |  |  |  | |  |
| **2** | bef-pat.  bef-asept.  aft-b.f.  aft-pat.  aft.p.surr. | | HR  HW  🌕 missed  🌕 gloves | **2** | bef-pat.  bef-asept.  aft-b.f.  aft-pat.  aft.p.surr. | | HR  HW  🌕 missed  🌕 gloves | **2** | bef-pat.  bef-asept.  aft-b.f.  aft-pat.  aft.p.surr. | | HR  HW  🌕 missed  🌕 gloves | **2** | bef-pat.  bef-asept.  aft-b.f.  aft-pat.  aft.p.surr. | | HR  HW  🌕 missed  🌕 gloves |
|  |  | |  |  |  | |  |  |  | |  |  |  | |  |
| **3** | bef-pat.  bef-asept.  aft-b.f.  aft-pat.  aft.p.surr. | | HR  HW  🌕 missed  🌕 gloves | **3** | bef-pat.  bef-asept.  aft-b.f.  aft-pat.  aft.p.surr. | | HR  HW  🌕 missed  🌕 gloves | **3** | bef-pat.  bef-asept.  aft-b.f.  aft-pat.  aft.p.surr. | | HR  HW  🌕 missed  🌕 gloves | **3** | bef-pat.  bef-asept.  aft-b.f.  aft-pat.  aft.p.surr. | | HR  HW  🌕 missed  🌕 gloves |
|  |  | |  |  |  | |  |  |  | |  |  |  | |  |
| **4** | bef-pat.  bef-asept.  aft-b.f.  aft-pat.  aft.p.surr. | | HR  HW  🌕 missed  🌕 gloves | **4** | bef-pat.  bef-asept.  aft-b.f.  aft-pat.  aft.p.surr. | | HR  HW  🌕 missed  🌕 gloves | **4** | bef-pat.  bef-asept.  aft-b.f.  aft-pat.  aft.p.surr. | | HR  HW  🌕 missed  🌕 gloves | **4** | bef-pat.  bef-asept.  aft-b.f.  aft-pat.  aft.p.surr. | | HR  HW  🌕 missed  🌕 gloves |
|  |  | |  |  |  | |  |  |  | |  |  |  | |  |
| **5** | bef-pat.  bef-asept.  aft-b.f.  aft-pat.  aft.p.surr. | | HR  HW  🌕 missed  🌕 gloves | **5** | bef-pat.  bef-asept.  aft-b.f.  aft-pat.  aft.p.surr. | | HR  HW  🌕 missed  🌕 gloves | **5** | bef-pat.  bef-asept.  aft-b.f.  aft-pat.  aft.p.surr. | | HR  HW  🌕 missed  🌕 gloves | **5** | bef-pat.  bef-asept.  aft-b.f.  aft-pat.  aft.p.surr. | | HR  HW  🌕 missed  🌕 gloves |
|  |  | |  |  |  | |  |  |  | |  |  |  | |  |
| **6** | bef-pat.  bef-asept.  aft-b.f.  aft-pat.  aft.p.surr. | | HR  HW  🌕 missed  🌕 gloves | **6** | bef-pat.  bef-asept.  aft-b.f.  aft-pat.  aft.p.surr. | | HR  HW  🌕 missed  🌕 gloves | **6** | bef-pat.  bef-asept.  aft-b.f.  aft-pat.  aft.p.surr. | | HR  HW  🌕 missed  🌕 gloves | **6** | bef-pat.  bef-asept.  aft-b.f.  aft-pat.  aft.p.surr. | | HR  HW  🌕 missed  🌕 gloves |
|  |  | |  |  |  | |  |  |  | |  |  |  | |  |
| **7** | bef-pat.  bef-asept.  aft-b.f.  aft-pat.  aft.p.surr. | | HR  HW  🌕 missed  🌕 gloves | **7** | bef-pat.  bef-asept.  aft-b.f.  aft-pat.  aft.p.surr. | | HR  HW  🌕 missed  🌕 gloves | **7** | bef-pat.  bef-asept.  aft-b.f.  aft-pat.  aft.p.surr. | | HR  HW  🌕 missed  🌕 gloves | **7** | bef-pat.  bef-asept.  aft-b.f.  aft-pat.  aft.p.surr. | | HR  HW  🌕 missed  🌕 gloves |
|  |  | |  |  |  | |  |  |  | |  |  |  | |  |
| **8** | bef-pat.  bef-asept.  aft-b.f.  aft-pat.  aft.p.surr. | | HR  HW  🌕 missed  🌕 gloves | **8** | bef-pat.  bef-asept.  aft-b.f.  aft-pat.  aft.p.surr. | | HR  HW  🌕 missed  🌕 gloves | **8** | bef-pat.  bef-asept.  aft-b.f.  aft-pat.  aft.p.surr. | | HR  HW  🌕 missed  🌕 gloves | **8** | bef-pat.  bef-asept.  aft-b.f.  aft-pat.  aft.p.surr. | | HR  HW  🌕 missed  🌕 gloves |

* To be completed by the data manager.

** **Optional**, to be used if appropriate, according to the local needs and regulations.

Revised August 2009

General Recommendations

(refer to the Hand Hygiene Technical Reference Manual)

1. In the context of open and direct observations, the observer introduces him/herself to the health-care worker and to the patient when appropriate, explains his/her task and proposes immediate informal feed back.
2. The health-care worker, belonging to one of the main four following professional categories (see below), is observed during the delivery of health-care activities to patients.
3. Detected and observed data should be recorded with a pencil in order to be immediately corrected if needed.
4. The top of the form (header) is completed before starting data collection (excepted end time and session duration).
5. The session should last no more than 20 minutes (± 10 minutes according to the observed activity); the end time and the session duration are to be completed at the end of the observation session.
6. The observer may observe up to three health-care workers simultaneously, if the density of hand hygiene opportunities permits.
7. Each column of the grid to record hand hygiene practices is intended to be dedicated to a specific professional category. Therefore numerous health-care workers may be sequentially included during one session in the column dedicated to their category. Alternatively each column may be dedicated to a single health-care worker only of whom the professional category should be indicated.
8. As soon as you detect an indication for hand hygiene, count an opportunity in the appropriate column and cross the square corresponding to the indication(s) you detected. Then complete all the indications that apply and the related hand hygiene actions observed or missed.
9. Each opportunity refers to one line in each column; each line is independent from one column to another.
10. Cross items in squares (several may apply for one opportunity) or circles (only a single item may apply at one moment).
11. When several indications fall in one opportunity, each one must be recorded by crossing the squares.
12. Performed or missed actions must always be registered within the context of an opportunity.
13. Glove use may be recorded only when the hand hygiene action is missed while the health-care worker is wearing gloves.

Short description of items

| **Facility:** | to complete according to the local nomenclature | | |
| --- | --- | --- | --- |
| **Service:** | to complete according to the local nomenclature | | |
| **Ward:** | to complete according to the local nomenclature | | |
| **Department:** | to complete according to the following standardized nomenclature: | | |
|  | medical, including dermatology, neurology, haematology, oncology, etc. | | surgery, including neurosurgery, urology, EENT, ophthalmology, etc. |
|  | mixed (medical & surgical), including gynaecology | | obstetrics, including related surgery |
|  | paediatrics, including related surgery | | intensive care & resuscitation |
|  | emergency unit | | long term care & rehabilitation |
|  | ambulatory care, including related surgery | | other (to specify) |
| **Period N°:** | 1) pre- / 2) post-intervention; and then according to the institutional counter. | | |
| **Date:** | day (dd) / month (mm) / year (yy) | | |
| **Start/end time:** | hour (hh) / minute (mm). | | |
| **Session duration:** | difference between start and end time, resulting in minutes of observation. | | |
| **Session N°:** | attributed at the moment of data entry for analysis. | | |
| **Observer:** | observer’s initials (the observer is responsible for the data collection and for checking their accuracy before submitting the form for analysis. | | |
| **Page N°:** | to write only when more than one form is used for one session. | | |
| **Prof.cat:** | according to the following classification: | | |
|  | **1. nurse / midwife** | 1.1 nurse, 1.2 midwife, 1.3 student. | |
|  | **2. auxiliary** |  | |
|  | **3. medical doctor** | 3.1 in internal medicine, 3.2 surgeon, 3.3 anaesthetist / resuscitator / emergency physician, 3.4 paediatrician, 3.5 gynaecologist, 3.6 consultant, 3.7 medical student. | |
|  | **4. other health-care worker** | 4.1 therapist (physiotherapist, occupational therapist, audiologist, speech therapist), 4.2 technician (radiologist, cardiology technician, operating room technician, laboratory technician, etc), 4.3 other (dietician, dentist, social worker and any other health-related professional involved in patient care), 4.4 student. | |
| **Number:** | number of observed health-care workers belonging to the same professional category (same code) as they enter the field of observation and you detect opportunities. | | |
| **Opp(ortunity):** | defined by one indication at least | | |
| **Indication:** | reason(s) that motivate(s) hand hygiene action; all indications that apply at one moment must be recorded | | |
|  | bef.pat: before touching a patient | | aft.b.f: after body fluid exposure risk |
|  | bef.asept: before clean/aseptic procedure | | aft.pat: after touching a patient |
|  |  | | aft.p.surr: after touching patient surroundings |
| **HH action:** | response to the hand hygiene indication(s); it can be either a positive action by performing handrub or handwash, or a negative action by missing handrub or handwash | | |
|  | HR: hand hygiene action by handrubbing with an alcohol-based formula  HW: hand hygiene action by handwashing with soap and water | | Missed: no hand hygiene action performed |

Observation Form – Basic Compliance Calculation

|  | **Facility:** | | | | | | | | **Period:** | | | | **Setting:** | | | | | |
| --- | --- | --- | --- | --- | --- | --- | --- | --- | --- | --- | --- | --- | --- | --- | --- | --- | --- | --- |
|  | **Prof.cat.** | | | | **Prof.cat.** | | | | **Prof.cat.** | | | | **Prof.cat.** | | | **Total per session** | | |
| **Session N°** | **Opp (n)** | **HW (n)** | **HR**  **(n)** | **Opp (n)** | | **HW (n)** | **HR**  **(n)** | **Opp (n)** | | **HW (n)** | **HR**  **(n)** | **Opp (n)** | | **HW (n)** | **HR**  **(n)** | **Opp (n)** | **HW (n)** | **HR**  **(n)** |
| **1** |  |  |  |  | |  |  |  | |  |  |  | |  |  |  |  |  |
| **2** |  |  |  |  | |  |  |  | |  |  |  | |  |  |  |  |  |
| **3** |  |  |  |  | |  |  |  | |  |  |  | |  |  |  |  |  |
| **4** |  |  |  |  | |  |  |  | |  |  |  | |  |  |  |  |  |
| **5** |  |  |  |  | |  |  |  | |  |  |  | |  |  |  |  |  |
| **6** |  |  |  |  | |  |  |  | |  |  |  | |  |  |  |  |  |
| **7** |  |  |  |  | |  |  |  | |  |  |  | |  |  |  |  |  |
| **8** |  |  |  |  | |  |  |  | |  |  |  | |  |  |  |  |  |
| **9** |  |  |  |  | |  |  |  | |  |  |  | |  |  |  |  |  |
| **10** |  |  |  |  | |  |  |  | |  |  |  | |  |  |  |  |  |
| **11** |  |  |  |  | |  |  |  | |  |  |  | |  |  |  |  |  |
| **12** |  |  |  |  | |  |  |  | |  |  |  | |  |  |  |  |  |
| **13** |  |  |  |  | |  |  |  | |  |  |  | |  |  |  |  |  |
| **14** |  |  |  |  | |  |  |  | |  |  |  | |  |  |  |  |  |
| **15** |  |  |  |  | |  |  |  | |  |  |  | |  |  |  |  |  |
| **16** |  |  |  |  | |  |  |  | |  |  |  | |  |  |  |  |  |
| **17** |  |  |  |  | |  |  |  | |  |  |  | |  |  |  |  |  |
| **18** |  |  |  |  | |  |  |  | |  |  |  | |  |  |  |  |  |
| **19** |  |  |  |  | |  |  |  | |  |  |  | |  |  |  |  |  |
| **20** |  |  |  |  | |  |  |  | |  |  |  | |  |  |  |  |  |
| **Total** |  |  |  |  | |  |  |  | |  |  |  | |  |  |  |  |  |
| **Calculation** | **Act (n) =**  **Opp (n) =** | | | **Act (n) =**  **Opp (n)** = | | | | | **Act (n) =**  **Opp (n) =** | | | **Act (n)** =  **Opp (n) =** | | | | **Act (n)** =  **Opp (n) =** | | |
| **Compliance** |  | | |  | | | | |  | | |  | | | |  | | |

Compliance (%) = Actions x 100

Opportunities

Instructions for use

1. Define the setting outlining the scope for analysis and report related data according to the chosen setting.
2. Check data in the observation form. Hand hygiene actions not related to an indication should not be taken into account and vice versa.
3. Report the session number and the related observation data in the same line. This attribution of session number validates the fact that data has been taken into count for compliance calculation.
4. Results per professional category and per session (vertical):

4.1 Sum up recorded opportunities (opp) in the case report form per professional category: report the sum in the corresponding cell in the calculation form.

4.2 Sum up the positive hand hygiene actions related to the total of opportunities above, making difference between handwash (HW) and handrub (HR): report the sum in the corresponding cell in the calculation form.

4.3 Proceed in the same way for each session (data record form).

4.4 Add up all sums per each professional category and put the calculation to calculate the compliance rate (given in percent)

1. The addition of results of each line permits to get the global compliance at the end of the last right column.

Observation form – Optional Calculation Form

(Indication-related compliance with hand hygiene)

|  | **Facility:** | | |  | | | **Period:** | | | **Setting:** | | |  | | |
| --- | --- | --- | --- | --- | --- | --- | --- | --- | --- | --- | --- | --- | --- | --- | --- |
|  | **Before touching a patient** | | | **Before clean/ aseptic procedure** | | | **After body fluid exposure risk** | | | **After touching a patient** | | | **After touching patient surroundings** | | |
| **Session N°** | **Indic (n)** | **HW (n)** | **HR**  **(n)** | **Indic (n)** | **HW (n)** | **HR**  **(n)** | **Indic (n)** | **HW (n)** | **HR**  **(n)** | **Indic (n)** | **HW (n)** | **HR**  **(n)** | **Indic (n)** | **HW (n)** | **HR**  **(n)** |
| **1** |  |  |  |  |  |  |  |  |  |  |  |  |  |  |  |
| **2** |  |  |  |  |  |  |  |  |  |  |  |  |  |  |  |
| **3** |  |  |  |  |  |  |  |  |  |  |  |  |  |  |  |
| **4** |  |  |  |  |  |  |  |  |  |  |  |  |  |  |  |
| **5** |  |  |  |  |  |  |  |  |  |  |  |  |  |  |  |
| **6** |  |  |  |  |  |  |  |  |  |  |  |  |  |  |  |
| **7** |  |  |  |  |  |  |  |  |  |  |  |  |  |  |  |
| **8** |  |  |  |  |  |  |  |  |  |  |  |  |  |  |  |
| **9** |  |  |  |  |  |  |  |  |  |  |  |  |  |  |  |
| **10** |  |  |  |  |  |  |  |  |  |  |  |  |  |  |  |
| **11** |  |  |  |  |  |  |  |  |  |  |  |  |  |  |  |
| **12** |  |  |  |  |  |  |  |  |  |  |  |  |  |  |  |
| **13** |  |  |  |  |  |  |  |  |  |  |  |  |  |  |  |
| **14** |  |  |  |  |  |  |  |  |  |  |  |  |  |  |  |
| **15** |  |  |  |  |  |  |  |  |  |  |  |  |  |  |  |
| **16** |  |  |  |  |  |  |  |  |  |  |  |  |  |  |  |
| **17** |  |  |  |  |  |  |  |  |  |  |  |  |  |  |  |
| **18** |  |  |  |  |  |  |  |  |  |  |  |  |  |  |  |
| **19** |  |  |  |  |  |  |  |  |  |  |  |  |  |  |  |
| **20** |  |  |  |  |  |  |  |  |  |  |  |  |  |  |  |
| **Total** |  |  |  |  |  |  |  |  |  |  |  |  |  |  |  |
| **Calculation** | **Act (n) =**  **Indic1 (n) =** | | | **Act (n) =**  **Indic2 (n) =** | | | **Act (n) =**  **Indic3 (n) =** | | | **Act (n) =**  **Indic4 (n) =** | | | **Act (n) =**  **Indic5 (n) =** | | |
| **Ratio**  **act / indic^🞱^** |  | | |  | | |  | | |  | | |  | | |

Instructions for use

1. Define the setting outlining the scope for analysis and report related data according to the chosen setting.
2. Check data in the observation form. Hand hygiene actions not related to an indication should not be taken into account and vice versa.
3. If several indications occur within the same opportunity, each one should be considered separately as well as the related action.
4. Report the session number and the related observation data in the same line. This attribution of session number validates the fact that data has been taken into count for compliance calculation.
5. Results per indication (indic) and per session (vertical):

4.1 Sum up indications per indication in the observation form: report the sum in the corresponding cell in the calculation form.

4.2 Sum up positive hand hygiene actions related to the total of indications above, making the difference between handwash (HW) and handrub (HR): report the sum in the corresponding cell in the calculation form.

4.3 Proceed in the same way for each session (observation form).

4.4 Add up all sums per each indication and put the calculation to calculate the ratio (given in percent)

**^🞱^Note:** This calculation is not exactly a compliance result, as the denominator of the calculation is an indication instead of an opportunity. Action is artificially overestimated according to each indication. However, the result gives an overall idea of health-care worker’s behaviour towards each type
of indication.
