# Supplementary material for: Effectiveness and sustainability of the WHO multimodal hand hygiene improvement strategy in the University Hospital Bouaké, Republic of Côte d'Ivoire in the context of the COVID-19 pandemic
Source: Antimicrob Resist Infect Control. 2022 Feb 17;11:36. doi: 10.1186/s13756-021-01032-4 (PMC8851710; doi:10.1186/s13756-021-01032-4)
Supplement: Supplementary file 3 — Additional file 3. WHO Ward Infrastructure Survey. [file 13756_2021_1032_MOESM3_ESM.doc]

Ward Infrastructure Survey

| Period Number* |  |
| --- | --- |

- The survey should be completed by the hand hygiene programme co-ordinator or an identified and informed health-care worker working within the ward (e.g. a senior nurse who can complete the survey while walking around the ward).
- This questionnaire is in two parts: 1) **questions** on handwashing and handrub facilities and resources available in the ward; 2) a **grid** to assess the exact number of hand hygiene resources and products in place, to be completed by walking to each room or area where patient care/treatment takes place (i.e. the point of care).
- **Short Glossary:**

**Alcohol-based handrub formulation:** an alcohol-containing preparation (liquid, gel or foam) designed for application to the hands to kill germs.

**Facility:** health-care setting where the survey is being carried out (e.g. hospital, ambulatory, long-term facility, etc).

**Handrubbing:** treatment of hands with an antiseptic handrub (alcohol-based formulation).

**Handwashing:** washing hands with plain or antimicrobial soap and water.

**Service:** a branch of a hospital staff that provides specified patient care.

**Ward:** a division, floor, or room of a hospital for a particular category or group of patients (it corresponds to the smallest segmentation of the health-care facility; one service can include multiple wards)**.**

| 1. Date: |  | 1. Facility: |  |
| --- | --- | --- | --- |
|  |  |  |  |
| 1. Ward: |  | 1. Service**: |  |
|  |  |  |  |
| 1. City** |  | 1. Country** |  |

1. Department (please select the department which best represents yours):

Internal medicine  Surgery  Intensive care unit  Mixed medical/surgical

Emergency unit  Obstetrics  Paediatrics  Long-term/rehabilitation

Outpatient clinic  Other

1. Position of the person completing this questionnaire:

Head nurse  Head physician  Hand hygiene programme co-ordinator

Hand hygiene programme deputy co-ordinator  Other infection control team member  Others

1. Number of health-care workers on this ward:       **Nurses**       **Physicians**       **Auxiliaries**
2. Is water regularly available?  **Always**  **Intermittently**  **Rarely**  **Never**

* To be completed by the data manager.

** **Optional**, to be used if appropriate, according to the local needs and regulations.

Revised August 2009

1. Is running water available?  **Yes**  **No**
2. Is water visibly clean?  **Yes**  **No**  **Don’t know**
3. What kind of taps is available?  **Hand-operated**  **Elbow/wrist-operated**

**Foot-operated**  **Automatic**

1. Are disposable towels available at all sinks?  **Always**  **Intermittently**  **Rarely**  **Never**
2. Is soap available at all sinks?  **Always**  **Intermittently**  **Rarely**  **Never**
3. Is an alcohol-based handrub available?  **Always**  **Intermittently**  **Rarely**  **Never**
4. If yes, what type of handrub dispensers are available? (select all applicable answers)

Pocket bottle  Bottle affixed to trolley/tray  Bottle affixed to bed

Wall dispenser  Dispenser located on bedside table/trolley

1. If wall dispensers are available, are they placed at the point of care*?

Yes Yes but not at each point of care No

1. Does every health-care worker have easy access to handrub pocket bottles?

**Always**  **Intermittently**  **Rarely**  **Never**  **Not applicable**

1. Is there an assigned person responsible for the refilling or replacement of empty dispensers?

**Yes**  **No**

1. Are handrub dispensers replaced when empty?

**Always**  **Intermittently**  **Rarely**  **Never**  **Not applicable**

1. Are posters illustrating handwash technique displayed beside each sink?  **Yes**  **No**
2. Are posters illustrating handrub technique displayed close to the dispensers and in multiple areas of the ward?  **Yes**  **No**
3. Are posters illustrating indications for hand hygiene displayed in multiple areas of the ward?

**Yes**  **No**

1. Is any other type of reminder on hand hygiene displayed/available on this ward?  **Yes**  **No**

1. Are examination gloves available on this ward?  **Always**   **Intermittently**  **Rarely**  **Never**
2. Are audits on hand hygiene compliance periodically performed on this ward?  **Yes**  **No**
3. If yes, how frequently?  **At least once a year**  **At least once every 2 years**  **Less frequently**

**Please now walk to each room or area where patient care/treatment takes place in this ward (i.e. the point of care*) and complete the table below.**

|  | Room N°/ID | Total N° of beds in this room/area | N° of beds with handrub within arm’s reach | N° of sinks in this room/area | N° of sinks with clean water | N° of sinks with soap | N° of sinks with disposable towel | N° of sinks with clean water, soap, disposable towel | Total N° of handrub dispensers in this room/area | N° of fully-functioning and filled dispensers | N° of health-care workers encountered | N° of health-care workers encountered with handrub bottle in their pocket |
| --- | --- | --- | --- | --- | --- | --- | --- | --- | --- | --- | --- | --- |
| A) Patient rooms on this ward | | | | | | | | | | | | |
| 1 |  |  |  |  |  |  |  |  |  |  |  |  |
| 2 |  |  |  |  |  |  |  |  |  |  |  |  |
| 3 |  |  |  |  |  |  |  |  |  |  |  |  |
| 4 |  |  |  |  |  |  |  |  |  |  |  |  |
| 5 |  |  |  |  |  |  |  |  |  |  |  |  |
| 6 |  |  |  |  |  |  |  |  |  |  |  |  |
| 7 |  |  |  |  |  |  |  |  |  |  |  |  |
| 8 |  |  |  |  |  |  |  |  |  |  |  |  |
| 9 |  |  |  |  |  |  |  |  |  |  |  |  |
| 10 |  |  |  |  |  |  |  |  |  |  |  |  |
| TOT | / |  |  |  |  |  |  |  |  |  |  |  |
| B) Treatment rooms (ambulatory, day hospital, etc.) | | | | | | | | | | | | |
| 1 |  |  |  |  |  |  |  |  |  |  |  |  |
| 2 |  |  |  |  |  |  |  |  |  |  |  |  |
| 3 |  |  |  |  |  |  |  |  |  |  |  |  |
| 4 |  |  |  |  |  |  |  |  |  |  |  |  |
| 5 |  |  |  |  |  |  |  |  |  |  |  |  |
| TOT | / |  |  |  |  |  |  |  |  |  |  |  |
| C) Corridors and other areas with points of care* | | | | | | | | | | | | |
| 1 |  |  |  |  |  |  |  |  |  |  |  |  |
| 2 |  |  |  |  |  |  |  |  |  |  |  |  |
| 3 |  |  |  |  |  |  |  |  |  |  |  |  |
| 4 |  |  |  |  |  |  |  |  |  |  |  |  |
| 5 |  |  |  |  |  |  |  |  |  |  |  |  |
| TOT | / |  |  |  |  |  |  |  |  |  |  |  |
| TOT |  |  |  |  |  |  |  |  |  |  |  |  |

TOT = total; N° = number

*Point of care: the place where three elements occur together: the patient, the health-care worker, and care or treatment involving contact with the patient and his surroundings.

Lines may be added according to the number of participants.
