# Supplementary material for: Effectiveness and sustainability of the WHO multimodal hand hygiene improvement strategy in the University Hospital Bouaké, Republic of Côte d'Ivoire in the context of the COVID-19 pandemic
Source: Antimicrob Resist Infect Control. 2022 Feb 17;11:36. doi: 10.1186/s13756-021-01032-4 (PMC8851710; doi:10.1186/s13756-021-01032-4)
Supplement: Supplementary file 5 — Additional file 5. WHO Perception Survey for Health-Care Workers. [file 13756_2021_1032_MOESM5_ESM.doc]

Perception Survey for Health-Care Workers

| Period Number* |  |
| --- | --- |

You are in direct contact with patients on a daily basis and this is why we are interested in your **opinion** on health care-associated infections and hand hygiene.

- It should take you about 10 minutes to complete this questionnaire.
- Each question has **one answer only**.
- Please read the questions carefully and then respond spontaneously. Your answers are anonymous and will be kept confidential.
- **Short Glossary:**

**Alcohol-based handrub formulation:** an alcohol-containing preparation (liquid, gel or foam) designed for application to the hands to kill germs.

**Facility:** health-care setting where the survey is being carried out (e.g., hospital, ambulatory, long-term facility, etc).

**Handrubbing:** treatment of hands with an antiseptic handrub (alcohol-based formulation).

**Handwashing:** washing hands with plain or antimicrobial soap and water.

**Service:** a branch of a hospital staff that provides specified patient care.

**Ward:** a division, floor, or room of a hospital for a particular category or group of patients (it corresponds to the smallest segmentation of the health-care facility; one service can include multiple wards)**.**

| 1. Personal ID**: |  | 1. Date: |  |
| --- | --- | --- | --- |
|  |  |  |  |
| 1. Facility: |  | 1. Service**: |  |
|  |  |  |  |
| 1. Ward**: |  | 1. City**: |  |
|  |  |  |  |
| 1. Country**: |  |  |  |
|  |  |  |  |

1. Gender:  **Female**  **Male**
2. Age (years): **15-19**  **20-24** **25-29** **30-34** **35-39** **40-44** **45-49** **50-54** **55-59** **60+**
3. Profession***:  **Nurse**  **Auxiliary nurse**   **Midwife**  **Medical doctor**  Resident

Technician  Therapist Nurse student  Medical student  Other

* To be completed by the data manager.

** **Optional**, to be used if appropriate, according to the local needs and regulations.

*****Technicians**: radiologist, cardiology technician, operating room technician, laboratory technician

**Therapist**: physiotherapist, occupational therapist, audiologist, speech therapist

**Other**: dietician, dentist, social worker, etc.

Revised August 2009

1. Department (please select the department which best represents yours):

Internal medicine  Surgery  Intensive care unit  Mixed medical/surgical

Emergency unit  Obstetrics  Paediatrics  Long-term/rehabilitation

Outpatient clinic  Other

1. Did you receive formal training in hand hygiene in the last three years?  **Yes**  **No**
2. Do you routinely use an alcohol-based handrub for hand hygiene?  **Yes**  **No**
3. In your opinion, what is the average percentage of hospitalised patients who will develop a health care-associated infection (between 0 and 100%)?

      %  I don't know

1. In general, what is the impact of a health care-associated infection on a patient's clinical outcome?

Very low  Low  High  Very high

1. What is the effectiveness of hand hygiene in preventing health care-associated infection?

Very low  Low  High  Very high

1. Among all patient safety issues, how important is hand hygiene at your institution?

Low priority  Moderate priority  High priority  Very high priority

1. On average, in what percentage of situations requiring hand hygiene do health-care workers in your hospital actually perform hand hygiene, either by handrubbing or handwashing (between 0 and 100%)?

      %  I don't know

1. In your opinion, how effective would the following actions be to improve hand hygiene permanently in your institution?

Please tick one “ ” on the scale according to your opinion.

1. Leaders and senior managers at your institution support and openly promote hand hygiene.

Not effective - - - - - - - - - - - - - - - - - -  Very effective

1. The health-care facility makes alcohol-based handrub always available at each point of care.

Not effective - - - - - - - - - - - - - - - - - -  Very effective

1. Hand hygiene posters are displayed at point of care as reminders.

Not effective - - - - - - - - - - - - - - - - - -  Very effective

1. Each health-care worker receives education on hand hygiene.

Not effective - - - - - - - - - - - - - - - - - -  Very effective

1. Clear and simple instructions for hand hygiene are made visible for every health-care worker.

Not effective - - - - - - - - - - - - - - - - - -  Very effective

1. Health-care workers regularly receive feedback on their hand hygiene performance.

Not effective - - - - - - - - - - - - - - - - - -  Very effective

1. You always perform hand hygiene as recommended (being a good example for your colleagues).

Not effective - - - - - - - - - - - - - - - - - -  Very effective

1. Patients are invited to remind health-care workers to perform hand hygiene.

Not effective - - - - - - - - - - - - - - - - - -  Very effective

1. What importance does the head of your department attach to the fact that you perform optimal hand hygiene?

No importance - - - - - - - - - - - - - - - - - -  Very high importance

1. What importance do your colleagues attach to the fact that you perform optimal hand hygiene?

No importance - - - - - - - - - - - - - - - - - -  Very high importance

1. What importance do patients attach to the fact that you perform optimal hand hygiene?

No importance - - - - - - - - - - - - - - - - - -  Very high importance

1. How do you consider the effort required by you to perform good hand hygiene when caring for patients?

No effort - - - - - - - - - - - - - - - - - -  A big effort

1. On average, in what percentage of situations requiring hand hygiene do you actually perform hand hygiene, either by handrubbing or handwashing (between 0 and 100%)?

      %

Thank you very much for your time!

Follow-Up Perception Survey for Health-Care Workers

| Period Number* |  |
| --- | --- |

You are in direct contact with patients on a daily basis and this is why we are interested in your **opinion** on health care-associated infections and hand hygiene.

- It should take you no more than 15 minutes to complete this questionnaire.
- Each question has **one answer only**.
- Please read the questions carefully and then respond spontaneously. Your answers are anonymous and will be kept confidential.
- This questionnaire is in two parts: **part 1** includes the same questions that you may have answered during the a previous evaluation period; **part 2** includes some additional questions to find out your opinion of the strategies and tools being currently used to promote hand hygiene at your institution.
- **Short Glossary:**

**Alcohol-based handrub formulation:** an alcohol-containing preparation (liquid, gel or foam) designed for application to the hands to kill germs.

**Facility:** health-care setting where the survey is being carried out (e.g., hospital, ambulatory, long-term facility, etc).

**Handrubbing:** treatment of hands with an antiseptic handrub (alcohol-based formulation).

**Handwashing:** washing hands with plain or antimicrobial soap and water.

**Service:** a branch of a hospital staff that provides specified patient care.

**Ward:** a division, floor, or room of a hospital for a particular category or group of patients (it corresponds to the smallest segmentation of the health-care facility; one service can include multiple wards).

Part 1

| 1. Personal ID**: |  | 1. Date: |  |
| --- | --- | --- | --- |
|  |  |  |  |
| 1. Facility: |  | 1. Service**: |  |
|  |  |  |  |
| 1. Ward**: |  | 1. City**: |  |
|  |  |  |  |
| 1. Country**: |  |  |  |

1. Gender:  **Female**  **Male**
2. Age:       **years**
3. Profession***:  **Nurse**  **Auxiliary nurse**   **Midwife**  **Medical doctor**  **Resident**

Technician  Therapist Nurse student Medical student  Other

* To be completed by the data manager

** **Optional**, to be used if appropriate, according to the local needs and regulations.

*****Technicians**: radiologist, cardiology technician, operating room technician, laboratory technician

**Therapist**: physiotherapist, occupational therapist, audiologist, speech therapist

**Others**: dietician, dentist, social worker, etc.

1. Department (please select the department which best represents yours):

Internal medicine  Surgery  Intensive care unit  Mixed medical/surgical

Emergency unit  Obstetrics  Paediatrics  Long-term/rehabilitation

Outpatient clinic  Other

1. Did you receive formal training in hand hygiene in the last three years?  **Yes**  **No**
2. Do you routinely use an alcohol-based handrub for hand hygiene?  **Yes**  **No**
3. According to your knowledge, what is the average percentage of hospitalised patients who will develop a health care-associated infection (between 0 and 100%)?

      %  I don't know

1. In general, what is the impact of a health care-associated infection on patient's clinical outcome?

Very low  Low  High  Very high

1. What is the effectiveness of hand hygiene in preventing health care-associated infection?

Very low  Low  High  Very high

1. Among all patient safety issues, how important is hand hygiene at your institution?

Low priority  Moderate priority  High priority  Very high priority

1. On average, in what percentage of situations requiring hand hygiene do health-care workers in your hospital actually perform hand hygiene, either by handrubbing or handwashing (between 0 and 100%)?

      %  I don't know

1. In your opinion, how effective would the following actions be to improve hand hygiene permanently in your institution?

Please tick one “ ” on the scale according to your opinion.

1. Leaders and senior managers at your institution support and openly promote hand hygiene.

Not effective - - - - - - - - - - - - - - - - - -  Very effective

1. The health-care facility makes alcohol-based handrub always available at each point of care.

Not effective - - - - - - - - - - - - - - - - - -  Very effective

1. Hand hygiene posters are displayed at point of care as reminders.

Not effective - - - - - - - - - - - - - - - - - -  Very effective

1. Each health-care worker receives education on hand hygiene.

Not effective - - - - - - - - - - - - - - - - - -  Very effective

1. Clear and simple instructions for hand hygiene are made visible for every health-care worker.

Not effective - - - - - - - - - - - - - - - - - -  Very effective

1. Health-care workers regularly receive feedback on their hand hygiene performance.

Not effective - - - - - - - - - - - - - - - - - -  Very effective

1. You always perform hand hygiene as recommended (being a good example for your colleagues).

Not effective - - - - - - - - - - - - - - - - - -  Very effective

1. Patients are invited to remind health-care workers to perform hand hygiene.

Not effective - - - - - - - - - - - - - - - - - -  Very effective

1. What importance does the head of your department attach to the fact that you perform optimal hand hygiene?

No importance - - - - - - - - - - - - - - - - - -  Very high importance

1. What importance do your colleagues attach to the fact that you perform optimal hand hygiene?

No importance - - - - - - - - - - - - - - - - - -  Very high importance

1. What importance do patients attach to the fact that you perform optimal hand hygiene?

No importance - - - - - - - - - - - - - - - - - -  Very high importance

1. How do you consider the effort required by you to perform good hand hygiene when caring for patients?

No effort - - - - - - - - - - - - - - - - - -  A big effort

1. On average, in what percentage of situations requiring hand hygiene do you actually perform hand hygiene, either by handrubbing or handwashing (between 0 and 100%)?

      %

Part 2

1. Has the use of an alcohol-based handrub made hand hygiene easier to practice in your daily work?

Not at all - - - - - - - - - - - - - - - - - -  Very important

1. Is the use of alcohol-based handrubs well tolerated by your hands?

Not at all - - - - - - - - - - - - - - - - - -  Very well

1. Did knowing the results of hand hygiene observation in your ward help you and your colleagues to improve your hand hygiene practices?

Not at all - - - - - - - - - - - - - - - - - -  Very much

1. Has the fact of being observed made you paying more attention to your hand hygiene practices?

Not at all - - - - - - - - - - - - - - - - - -  Very much

1. Were the educational activities that you participated in important to improve your hand hygiene practices?

Not at all - - - - - - - - - - - - - - - - - -  Very important

1. Do you consider that the administrators in your institution are supporting hand hygiene improvement?

Not at all - - - - - - - - - - - - - - - - - -  Very much

1. Has the improvement of the safety climate (if actually improved in your institution as a result of the recent implementation of the hand hygiene promotion strategy) helped you personally to improve your hand hygiene practices?

Not at all - - - - - - - - - - - - - - - - - -  Very much

1. Has your awareness of your role in preventing health-care-associated infection by improving your hand hygiene practices increased during the current hand hygiene promotional campaign?

Not at all - - - - - - - - - - - - - - - - - -  Very much

Thank you very much for your time!
