# Supplementary material for: Effectiveness and sustainability of the WHO multimodal hand hygiene improvement strategy in the University Hospital Bouaké, Republic of Côte d'Ivoire in the context of the COVID-19 pandemic
Source: Antimicrob Resist Infect Control. 2022 Feb 17;11:36. doi: 10.1186/s13756-021-01032-4 (PMC8851710; doi:10.1186/s13756-021-01032-4)
Supplement: Supplementary file 6 — Additional file 6. Hand Hygiene Compliance at Baseline and Follow-up. [file 13756_2021_1032_MOESM6_ESM.docx]

| **Supplement Table 1.** Hand Hygiene Compliance at Baseline and Follow-up | | | | | | | | |  |  |  |  |  |
| --- | --- | --- | --- | --- | --- | --- | --- | --- | --- | --- | --- | --- | --- |
| Variable | | Baseline | | | 1^st^ Follow-up | | | P** | 2^nd^ Follow-up | | | P** | P*** |
|  |  | No. of HH actions | No. (%) of HH opportunities | Compliance,  % (95% CI)* | No. of HH actions | No. (%) of HH opportunities | Compliance,  % (95% CI)* |  | No. of HH actions | No. (%) of HH opportunities | Compliance,  % (95% CI)* |  |  |
| Overall | | 91 | 719 | 12.7 (7.8-17.5) | 339 | 921 | 36.8 (30.6-43.0) | <0.001 | 304 | 835 | 36.4 (29.9-42.9) | <0.001 | 0.902 |
| Professional category | |  |  |  |  |  |  |  |  |  |  |  |  |
|  | Medical Doctors | 21 | 164 (22.8) | 12.8 (2.5-23.1) | 94 | 279 (30.3) | 33.7 (22.6-44.8) | 0.001 | 116 | 296 (35.4) | 40.6 (29.2-52.0) | <0.001 | 0.232 |
|  | Nurses | 21 | 125 (17.4) | 16.8 (3.6-30.0) | 76 | 194 (21.1) | 39.2 (25.4-52.9) | 0.003 | 38 | 97 (11.6) | 39.2 (19.6-58.7) | 0.008 | 1.000 |
|  | Midwife | 3 | 54 (7.5) | 5.6 (-8.4-19.6) | 14 | 34 (3.7) | 41.2 (7.6-74.8) | 0.004 | 39 | 134 (16.0) | 29.1 (13.7-44.5) | 0.013 | 0.339 |
|  | Auxiliary Nurse | 18 | 155 (21.6) | 11.6 (1.5-21.7) | 100 | 267 (29.0) | 37.5 (25.8-49.1) | <0.001 | 63 | 191 (22.9) | 33.0 (19.6-46.4) | 0.001 | 0.486 |
|  | Others | 28 | 221 (30.7) | 12.7 (3.9-21.5) | 55 | 147 (16.0) | 37.4 (21.7-53.1) | <0.001 | 48 | 127 (15.2) | 37.8 (20.9-54.7) | <0.001 | 0.963 |
| Indication | |  |  |  |  |  |  |  |  |  |  |  |  |
|  | Bef. patient contact | 16 | 302 (42.0) | 5.3 (0.24-10.4) | 58 | 374 (40.6) | 15.5 (8.2-22.9) | 0.003 | 59 | 290 (34.7) | 20.3 (11.1-29.6) | <0.001 | 0.251 |
|  | Bef. aseptic task | 1 | 56 (7.8) | 1.8 (-5.2-8.8) | 16 | 103 (11.2) | 15.5 (1.5-29.6) | 0.058 | 28 | 145 (17.4) | 19.3 (6.42-32.2) | 0.025 | 0.588 |
|  | Aft. body fluid exposure risk | 2 | 13 (1.8) | 15.4 (-25.4-56.2) | 29 | 37 (4.0) | 78.4 (51.5-105.3) | 0.004 | 37 | 39 (4.7) | 94.9 (80.8-108.9) | <0.001 | 0.133 |
|  | Aft. Pat. contact | 65 | 241 (33.5) | 27.0 (15.7-38.2) | 193 | 305 (33.1) | 63.3 (52.4-74.1) | <0.001 | 124 | 232 (27.8) | 53.4 (40.6-66.3) | <0.001 | 0.105 |
|  | Aft. contact with pat. surroundings | 7 | 107 (14.9) | 6.5 (-2.9-16.0) | 43 | 102 (11.1) | 42.2 (22.9-61.4) | <0.001 | 56 | 129 (15.4) | 43.4 (26.2-60.6) | <0.001 | 0.892 |
| *width of CI adjusted for lack of independence by inflating standard error by a factor of 2. | | | | | | | | | | | | | |
| ** determined by Chi² test to reference baseline with standard error corrected by factor 2 to adjust for lack of independence  *** determined by Chi² test to first follow-up with standard error corrected by factor 2 to adjust for lack of independence | | | | | | | | | | | | | |
